# Enhancement of cranial nerves in Lyme neuroborreliosis: incidence and correlation with clinical symptoms and prognosis

**DOI:** 10.1007/s00234-022-02957-2

**Published:** 2022-05-24

**Authors:** Elisabeth S. Lindland, Anne Marit Solheim, Muhammad Nazeer Dareez, Randi Eikeland, Unn Ljøstad, Åse Mygland, Harald Reiso, Åslaug R. Lorentzen, Hanne F. Harbo, Mona K. Beyer

**Affiliations:** 1 Department of Radiology, Sorlandet Hospital, Sykehusveien 1, N-4809 Arendal, Norway; 2 Division of Radiology and Nuclear Medicine, Oslo University Hospital, Oslo, Norway; 3 Institute of Clinical Medicine, Oslo University Hospital, Oslo, Norway; 4 Department of Neurology, Sorlandet Hospital, Kristiansand, Norway; 5 Institute of Clinical Medicine, University of Bergen, Bergen, Norway; 6 The Norwegian National Advisory Unit on Tick-borne Diseases, Sorlandet Hospital, Kristiansand, Norway; 7 Faculty of Health and Sport Sciences, University of Agder, Kristiansand, Norway; 8 Department of Habilitation, Sorlandet Hospital, Kristiansand, Norway; 9 Department of Neurology, Oslo University Hospital, Oslo, Norway

**Keywords:** Lyme neuroborreliosis, Cranial nerves, Magnetic resonance imaging, Central nervous system infections, Vector borne diseases

## Abstract

**Purpose:**

Symptoms of cranial neuritis are a common presentation of Lyme neuroborreliosis (LNB). Imaging studies are scarce and report contradictory low prevalence of enhancement compared to clinical studies of cranial neuropathy. We hypothesized that MRI enhancement of cranial nerves in LNB is underreported, and aimed to assess the prevalence and clinical impact of cranial nerve enhancement in early LNB.

**Methods:**

In this prospective, longitudinal cohort study, 69 patients with acute LNB were examined with MRI of the brain. Enhancement of cranial nerves III–XII was rated. MRI enhancement was correlated to clinical findings of neuropathy in the acute phase and after 6 months.

**Results:**

Thirty-nine of 69 patients (57%) had pathological cranial nerve enhancement. Facial and oculomotor nerves were most frequently affected. There was a strong correlation between enhancement in the distal internal auditory canal and parotid segments of the facial nerve and degree of facial palsy (gamma = 0.95, *p* < .01, and gamma = 0.93, *p* < .01), despite that 19/37 nerves with mild-moderate enhancement in the distal internal auditory canal segment showed no clinically evident palsy. Oculomotor and abducens nerve enhancement did not correlate with eye movement palsy (gamma = 1.00 and 0.97, *p* = .31 for both). Sixteen of 17 patients with oculomotor and/or abducens nerve enhancement had no evident eye movement palsy.

**Conclusions:**

MRI cranial nerve enhancement is common in LNB patients, but it can be clinically occult. Facial and oculomotor nerves are most often affected. Enhancement of the facial nerve distal internal auditory canal and parotid segments correlate with degree of facial palsy.

**Supplementary Information:**

The online version contains supplementary material available at 10.1007/s00234-022-02957-2.

## Introduction

Lyme borreliosis is estimated to affect more than 200,000 Europeans and 470,000 in the USA each year [1, 2]. It is a tick-borne spirochetal infection that affects the nervous system in 10–15% of cases [3]. Most patients experience painful radiculoneuritis, and additional symptoms of cranial neuritis is a common clinical presentation in Lyme neuroborreliosis (LNB) [4, 5]. Cranial nerve involvement is reported to occur in 43% of LNB patients, and in 50% among those hospitalized [6, 7]. Facial nerve palsy accounts for up to 95% of these, while ocular motor nerve palsies constitute most of the remainder [4, 6, 7]. Data in a systematic review estimate that persistent cranial neuropathy after treatment affects 4–10% of LNB patients [8].

Imaging evaluation of cranial nerve enhancement together with laboratory tests are important in the work-up of patients with cranial nerve palsies. Findings of neoplasms, infections, demyelination, granulomatosis, or stroke have implications for treatment [9, 10]. When interpreting such images, one needs to be aware of physiological or spontaneous cranial nerve enhancement: The geniculate ganglion, mastoid, and tympanic segments of the facial nerve enhance in most individuals without facial nerve pathology [11, 12].

Several case reports on cranial nerve enhancement due to LNB exist [3, 13–17]. However, there are few imaging studies, and they report low numbers of cranial nerve enhancement compared to clinical reports. In a retrospective study from 2009, three of the 63 LNB patients who were examined with contrast had meningeal or cranial nerve enhancement [18]. Recent studies that only used clinical radiology reports of contrast enhanced brain examinations, found cranial nerve enhancement in one of 79 LNB patients and five of 29 LNB patients with facial palsy [19, 20]. Due to suboptimal designs of these studies with no description or validation of the enhancement ratings, and contradictory low prevalence of enhancement compared to clinical studies of cranial neuropathy, we hypothesized that enhancement of cranial nerves in LNB is underreported.

We aimed to describe the prevalence of enhancement of cranial nerves III–XII at the time of diagnosis of LNB, and to assess if enhancement is associated with clinical manifestation of neuropathy in the acute phase and 6 months after treatment.

## Materials and methods

### Study population

**Fig. 1:**
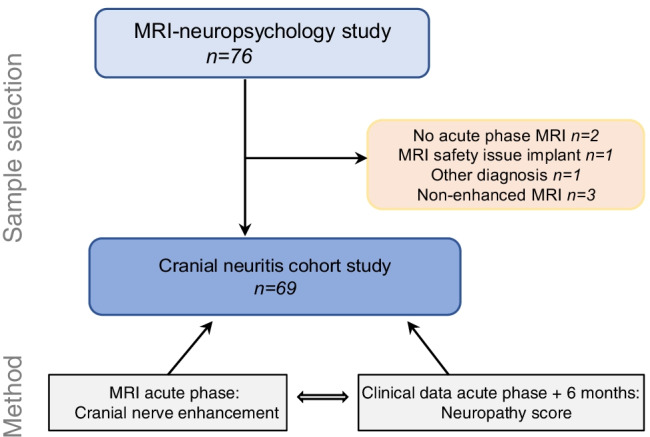
Flow chart of sample and data selection for this cranial neuritis cohort study. Patients with newly diagnosed Lyme neuroborreliosis were invited to join a non-inferiority randomized clinical oral treatment trial and a longitudinal case–control study with MRI and neuropsychology markers in the same research project (BorrSci). Patients who received intravenous treatment were also invited in the MRI-neuropsychology study. Invitation was declined by 13 eligible patients

This study is part of a research project (BorrSci) to improve knowledge on diagnostics and treatment of Lyme Borreliosis. It is a sub study of the participants included in a case–control study to investigate MRI markers and neurocognitive profile in LNB. The majority (63/76) also participated in a non-inferiority randomized clinical trial (RCT) with 6 versus 2 weeks oral doxycycline treatment [21]. The inclusion period was December 1st, 2015–December 18th, 2018. Patients were invited within 1 month after diagnosis of LNB according to the European Federation of Neurological Societies’ (EFNS) diagnostic guidelines [22]. These guidelines state that LNB diagnosis is definite when all three of the following criteria are present, and possible in cases with two of the three criteria fulfilled: (1) Neurological symptoms suggestive of LNB without other obvious reasons, (2) cerebrospinal fluid pleocytosis (≥ 5 leukocytes/mm^3^), and (3) intrathecal *Borrelia burgdorferi* (*Bb*) antibody production. Exclusion criteria were age less than 18 years, pregnancy, breast feeding, implant with MRI safety issues, and severe claustrophobia. Figure 1 shows the selection of sample subjects for this cranial neuritis sub study.

### Imaging

Within 1 month after diagnosis and treatment initiation, 57 subjects were scanned on a Siemens Skyra 3 T and 12 subjects on a General Electric Signa 3 T. Parameters for the whole brain 3D sagittal T1 weighted and fat saturated sequence are provided in Table 1. Images for this study were acquired 10 min after intravenous injection of 0.2 ml/kg of 0.5 mmol/ml gadoterate meglumine (Guerbet, France).

**Table 1:** Magnetic resonance imaging parameters for whole brain 3D sagittal contrast enhanced T1 weighted and fat saturated sequence used to rate enhancement of cranial nerves III–XII

	Siemens Skyra 3 T (*n* = 57)	General Electric Signa 3 T (*n* = 12)
Description	T1_sag_space_FS_CE	SagCUBET1FatsatK +
Coil configuration	64 channel head	32 channel head
Slice thickness (mm)	0.93	1.0
FOV read (mm)	256	256
FOV phase	100%	100%
TR (ms)	500	650
TE (ms)	3.8	Minimum
Fat suppression	Fat sat	Fat sat
Band width (Hz/Px)	630	625
Turbo factor	40	30
Averages/NEX	1.0	1.0
Shim	B0 standard/B1 Trueform	Auto

### Enhancement rating

**Fig. 2:**
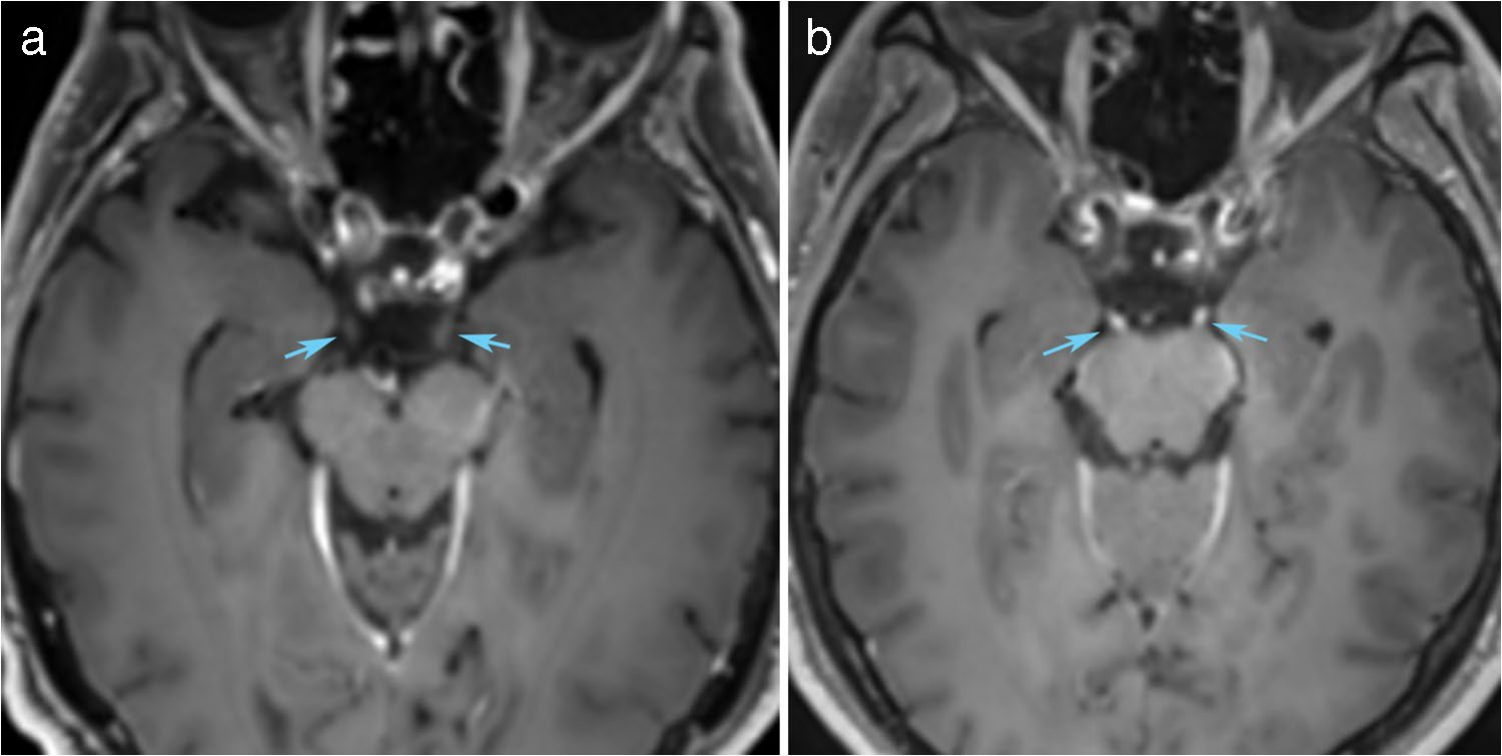
Demonstration of no versus strong enhancement of the oculomotor nerves on 1 mm axial image reconstruction from 3D T1-weighted and fat-suppressed acquisition. The subject in (**a**) demonstrates no enhancement of oculomotor nerves (arrows), while subject in (**b**) shows bilateral strong enhancement (arrows)

**Fig. 3:**
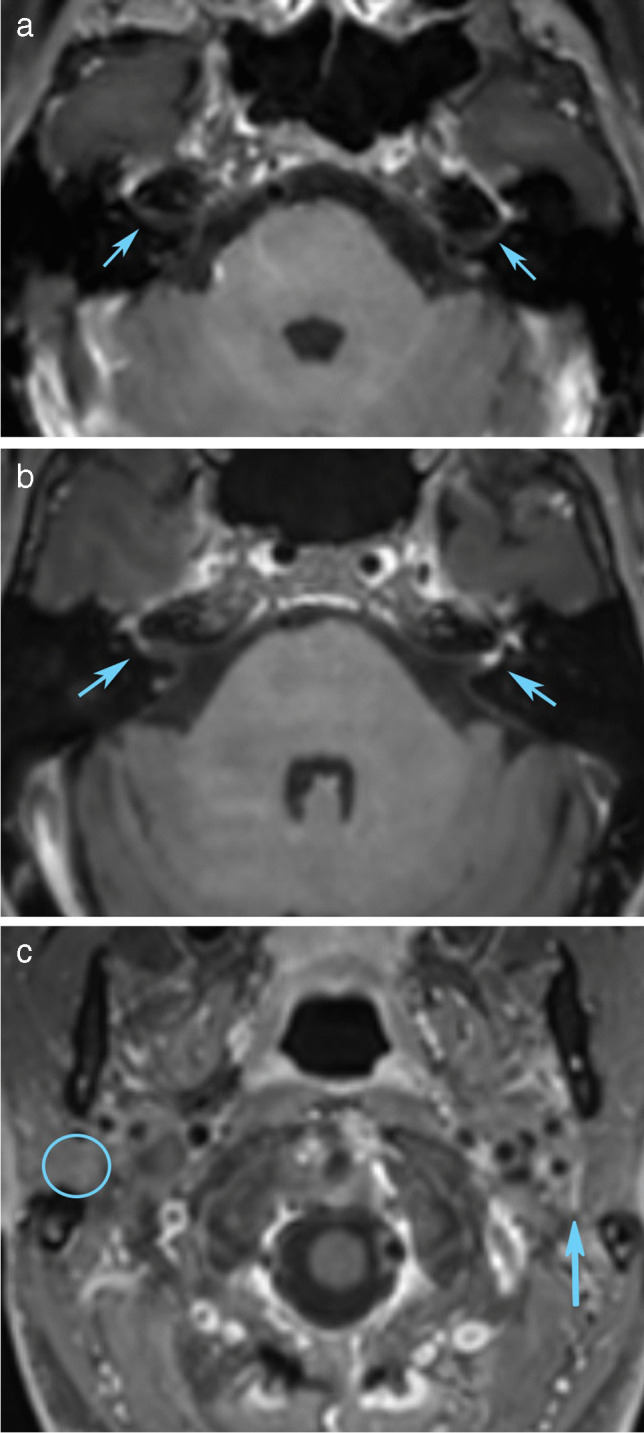
Evaluation of the facial nerve is complex due to a long course where some segments have physiological enhancement. Axial reconstruction from 3D T1-weighted and fat-suppressed acquisition shows no enhancement of the distal IAC segments (arrows in **a**), also there is no enhancement in the narrow labyrinthine segments that curve anterior toward the normal enhancing geniculate ganglions seen in the same axial slice. Axial images (**b** and **c**) are from a different subject at the level of the distal IAC and parotid gland, respectively. They demonstrate mild-moderate enhancement in the right distal IAC and strong enhancement in the left distal IAC (arrows in **b**), while no enhancement is seen in the right parotid segment (circle in **c**) and strong enhancement is present in the left parotid segment where the nerve is depicted as it curves anterior to approach the pes anserinus (arrow in **c**)

Enhancement of cranial nerves III–XII was rated by two neuroradiologists. An ordinal scale with four categories was used: No, possible, mild-moderate, and strong enhancement. The raters were blinded to the clinical records and each other’s ratings. The nerve segments that were rated were the cisternal segments, but for the facial nerve also the intraaxial, proximal internal auditory canal (IAC), distal IAC, labyrinthine, geniculate ganglion, tympanic, mastoid and parotid segments were rated, and cranial nerves IX–XI were jointly evaluated. Figures 2 and 3 show examples of enhancement ratings in important locations.

### Clinical rating

Clinical manifestation of neuropathy was evaluated by neurologists engaged in the clinical trial of the BorrSci project, at the start of treatment and six months after treatment. A three-point scale, also applied in a previous RCT [23], was used for clinically evident neuropathy: no manifestation (score 0), mild or moderate manifestation without influence on daily life function (score 1), and severe manifestation with influence on daily life function (score 2). For facial palsy, the mild or moderate degree was equivalent to the House-Brackman scale grade 1 and 2 with minimal symptoms at rest [24].

### Statistics

Statistical analyses were executed in IBM SPSS statistics version 25. Reproducibility of ratings was evaluated with percentage of agreement and weighted Cohen’s kappa [25]. Goodman and Kruskal’s gamma [26] was used to study degree of association between baseline enhancement in the facial nerve segments and facial palsy at baseline and six months after treatment, as well as oculomotor and abducens nerve enhancement and eye muscle palsy. Receiver operating characteristic (ROC) curves were used to study sensitivity and specificity for the usefulness of enhancement in selected facial nerve segments to predict the clinical manifestation of facial palsy [27]. *P* value of 0.05 was chosen as the significance level.

## Results

### Study population

A total of 69 subjects were analyzed, sample selection is shown in Fig. 1. There were 34 men and 35 women with mean age (range) 56.6 (27–78) and 58.6 (21–82) years, respectively.

Demographic, clinical, and laboratory characteristics are listed in Table 2. According to EFNS’ guidelines, there were 54 subjects with definite LNB in this patient cohort and 15 possible LNB cases [22]. Clinical neuropathy rating was not available for one subject at baseline, and seven subjects at 6 months (did not attend follow-up), they were not excluded from analyses. Mean interval between lumbar puncture leading to diagnosis of LNB and MRI examination was 16 (SD 8, range 2–31) days.

**Table 2:** Demographic, clinical, and laboratory characteristics for the sample subjects. Patients were included within one month after diagnosis of Lyme neuroborreliosis (35 women and 34 men)

Characteristic	Mean (standard deviation, range) or percentage (proportion)
Age (years)	57.6 (13.5, 21–82)
Duration of neurological symptoms (days)	28 (33, 1–180)
Tick-bite within last six months	58.7% (37/63)
Erythema migrans within last six months	24.6% (16/65)
Eye movement palsy	1.4% (1/69)
Facial palsy	53.6% (37/69)
Facial sensory disturbance	11.6% (8/69)
Reduced hearing	0
CSF^a^ cell count (cells/mm^3^)	188 (173, 7–752)
CSF^a^ protein level (g/l)	1.4 (0.8, 0.3–3.9)
Positive *Bb*^b^ antibody index	80.6% (54/67^c^)

^a^*CSF* cerebrospinal fluid

^b^*Bb Borrelia Burgdorferi*

^c^Two patients missed *Bb* antibody index due to blood contamination, both had positive *Bb* IgG in the cerebrospinal fluid and tick bite in the last six months. The subjects with negative index had duration of symptoms 15 (SD 14) days, and five had tick bite and two erythema migrans in the last 6 months

### Imaging and enhancement rating

Overall, 39 of 69 subjects (57%) had pathological cranial nerve enhancement and 13 cases had enhancement of multiple cranial nerves. Twenty-six subjects had enhancement of one cranial nerve, irrespective of unilateral or bilateral disease. Seven subjects had enhancement of two nerves, and six subjects had more than two nerves affected. The combinations of cranial nerve involvement for those with multiple nerve enhancement were five with C.N. III + VII, three with C.N. III, VI + VII, one with C.N. V + VII, one with C.N. VI + VII, one with C.N. III, V, VI + VII, and two with C.N. III + V–XII.

**Fig. 4:**
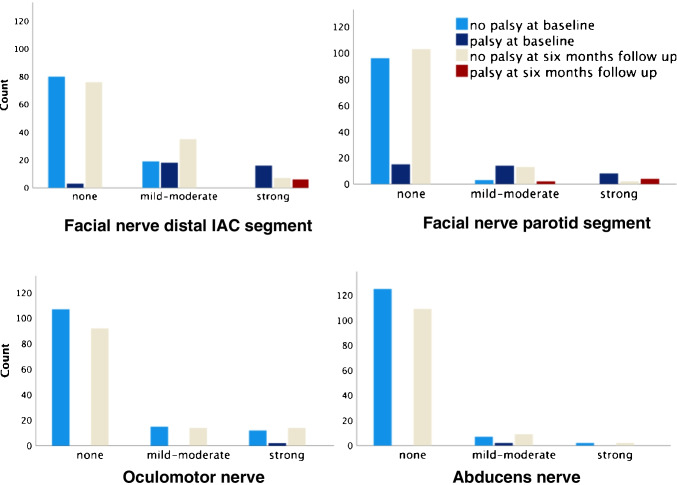
Bar charts provide a graphical summary of the main study findings. The bars are number of nerves for each enhancement degree (right and left side for each subject, possible enhancement was considered as no enhancement), clustered by palsy or no palsy at baseline and follow up. Facial palsy is in the top row, and eye movement palsy in the bottom row

Figure 4 provides a graphical summary of the degree of enhancement and clinical finding of palsy for the important nerve locations. Enhancement ratings for all nerves rated are provided in Table 3. Percentage of agreement and weighted kappa for each nerve location is provided in supplemental Table 1. The agreement was substantial (kappa = 0.61–0.80) for oculomotor, trigeminal, abducens, and labyrinthine segment of the facial nerve, and almost perfect (kappa = 0.81–1.00) for distal IAC and parotid segments of the facial nerve [28].

**Table 3:** Frequencies of ratings for the intensity of contrast enhancement for the nerve locations. The bottom row shows the number of subjects with bilateral enhancement. Enhancement of the geniculate ganglion, tympanic and mastoid segments of the facial nerve is a physiological finding

Enhancement^a^	Cranial nerves ^b^															
**Right**	III	IV	V	VI	VIIintra	VIIcist	VIIprox	VIIdist	VIIlab	VIIgen	VIItymp	VIImast	VIIpar	VIII	IX–XI	XII
None	52	69	65	62	68	67	68	39	37	0	0	2	56	66	67	67
Possible	1	0	0	1	1	0	0	2	3	0	1	3	0	1	0	0
Mild-moderate	9	0	4	5	0	1	1	20	24	51	64	62	10	2	2	2
Strong	7	0	0	1	0	1	0	8	5	18	4	2	3	0	0	0
**Left**	III	IV	V	VI	VIIintra	VIIcist	VIIprox	VIIdist	VIIlab	VIIgen	VIItymp	VIImast	VIIpar	VIII	IX–XI	XII
None	54	69	66	61	69	67	68	39	36	0	0	3	57	67	67	67
Possible	2	0	0	3	0	0	0	3	5	0	1	4	0	0	0	0
Mild-moderate	6	0	1	4	0	2	1	19	24	56	67	61	7	2	2	2
Strong	7	0	2	1	0	0	0	8	4	13	1	1	5	0	0	0
**Bilateral**	13	0	3	4	0	2	1	21	21	69	68	62	7	2	2	2

^a^For cases with different ratings by the neuroradiologists (295 of 2208 nerve locations), the joined score was set by the following algorithm: if one rated possible enhancement, the joined score was set to the other rater’s score; if ratings were slight-moderate and strong in the geniculate ganglion, tympanic or mastoid segments the joined score was set to mild-moderate. Other discrepancies were given a joined score by consensus (63 locations)

^b^Cranial nerves denoted with roman numerals. Abbreviations of C.N. VII segments: intra—intraaxial; cist—cisternal; prox—proximal internal auditory canal; dist—distal internal auditory canal; lab—labyrinthine; gen—geniculate ganglion; tymp—tympanic; mast—mastoid; par—parotid. All other nerves were rated in the cisternal segment only

### Facial palsy and nerve enhancement

There was a strong correlation between facial palsy grade at both baseline and six months and enhancement rate in the distal IAC (baseline gamma = 0.95, *p* < 0.01; 6 months gamma = 1.00, *p* = 0.01) and parotid segments (baseline gamma = 0.93, *p* < 0.01; 6 months gamma = 0.98, *p* = 0.01). Correlation between acute phase enhancement rate and facial palsy grade at baseline and 6 months for each of the facial nerve segments is provided in supplemental Table 2. Only the enhancement of distal IAC and parotid segments were included in further analyses with clinical data.

The proportion of palsy in facial nerves with acute phase enhancement of distal IAC segment was 34/53 (64%) at baseline and 6/48 (13%) at 6 months, and for the parotid segment 22/25 (88%) at baseline and 6/21(29%) at 6 months. At 6 months, 6/13 with strong distal IAC segment enhancement had persistent palsy and 4/6 with strong parotid segment enhancement had persistent palsy. Cross tables for enhancement rates of these segments versus palsy grade are presented in Table 4. Some nuances in these cross tables are not apparent in the gamma correlation coefficient: Firstly, a mild or moderate degree of enhancement has a weaker association with clinical facial palsy, especially in the distal IAC segment where 19/37 nerves had no associated baseline palsy. Secondly, when enhancement is present in the parotid segment, this per se may have a stronger association with the degree of palsy than the enhancement in the distal IAC segment, but the baseline gamma correlation coefficient is reduced by more subjects with no enhancement and some degree of palsy compared to distal IAC segment (14% among those with no parotid segment enhancement had baseline palsy versus 3% among those with no distal canal enhancement). ROC analysis was performed to elucidate these nuances, and showed that parotid enhancement is more specific, but less sensitive compared to distal IAC enhancement in predicting if facial palsy is present or not, both at baseline and after 6 months.

**Table 4:** Frequencies of acute phase enhancement rates in the distal IAC and parotid segments of the facial nerve, and facial palsy grade at baseline and six months after treatment (right and left side for each subject)

		Baseline^a^			Six months after treatment^a^			
		No palsy	Mild palsy	Severe palsy	No palsy	Mild palsy	Severe palsy	
Distal IAC^b^ enhancement	No enhancement	76	2	0	71	0	0	
	Possible	4	1	0	5	0	0	
	Mild-moderate	19	10	8	35	0	0	
	Strong	0	3	13	7	3	3	
	*Total*	*99*	*16*	*21*	*118*	*3*	*3*	
Parotid enhancement	No enhancement	96	9	6	103	0	0	
	Possible	0	0	0	0	0	0	
	Mild-moderate	3	7	7	13	1	1	
	Strong	0	0	8	2	2	2	
	*Total*	*99*	*16*	*21*	*118*	*3*	*3*	

^a^No clinical neuropathy rating for one subject at baseline and seven subjects at 6 months

^b^*IAC* internal auditory canal

**Fig. 5:**
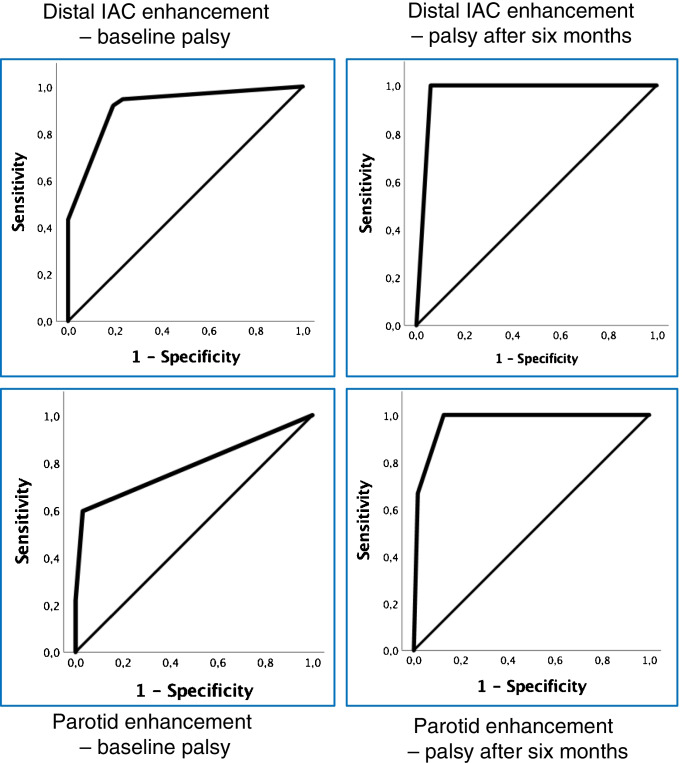
Receiver operating characteristic curves for enhancement rate show that parotid enhancement is more specific, but less sensitive compared to distal IAC enhancement in predicting if facial palsy is present or not, both at baseline and after 6 months

The ROC curves for the distal IAC (baseline AUC = 0.91, *p* < 0.01; 6 months AUC 0.97, *p* < 0.01) and parotid (baseline AUC = 0.79, *p* < 0.01; 6 months AUC 0.97, *p* < 0.01) segments are provided in Fig. 5.

### Eye movement palsy and nerve enhancement

Seventeen subjects had enhancement of oculomotor and/or abducens nerves in the acute phase. One of them had mild eye movement palsy at baseline, none after six months. Cross tables for enhancement rates of these nerves versus eye movement palsy grade are provided in Table 5. Correlation between enhancement rate and eye movement palsy was not significant (baseline gamma for C.N. III was 1.00, *p* = 0.31 and for C.N. VI gamma = 0.97, *p* = 0.31).

**Table 5:** Frequencies of enhancement rates for oculomotor and abducens nerves and eye movement palsy grade at baseline and six months after treatment (right and left side for each subject). No subjects had enhancement of the trochlear nerves

		Baseline^a^			Six months after treatment^a^		
		No palsy	Mild palsy	Severe palsy	No palsy	Mild palsy	Severe palsy
Oculomotor nerve enhancement	No enhancement	104	0	0	92	0	0
	Possible	3	0	0	3	0	0
	Mild-moderate	15	0	0	15	0	0
	Strong	12	2	0	14	0	0
	*Total*	*134*	*2*	*0*	*124*	*0*	*0*
Abducens nerve enhancement	No enhancement	121	0	0	109	0	0
	Possible	4	0	0	4	0	0
	Mild-moderate	7	2	0	9	0	0
	Strong	2	0	0	2	0	0
	*Total*	*134*	*2*	*0*	*124*	*0*	*0*

^a^No clinical neuropathy rating for one subject at baseline and seven subjects at 6 months

### Other cranial nerve symptoms and enhancement

Four subjects had trigeminal nerve enhancement, three of them had no facial sensory disturbance.

No subjects reported reduced hearing, two had enhancement of vestibulocochlear nerves.

## Discussion

In this large cohort study, we found that MRI cranial nerve enhancement occurs in more than half of LNB patients (57%, 39/69), and it may be clinically occult. Facial and oculomotor nerves are most often affected, and bilateral involvement is common. A fair proportion (13/69) have multiple cranial nerves affected. This supports our hypothesis that such imaging findings are underreported in previous studies, where some based their data on clinical radiology reports rather than visual evaluation. Cranial nerve enhancement was reported in 1–17%, the highest proportion was among LNB patients with facial palsy [18–20]. Advances in imaging techniques with 3 T, 3D acquisition, and fat suppression with increased sensitivity to enhancement, together with progress in radiology training, are possible explanations for the lower proportions of enhancement in earlier studies.

### Facial nerve

The enhancement pattern in the facial nerve resembles that of idiopathic facial palsy with main involvement of distal IAC and labyrinthine segments [29–31], and this suggests a common neurotoxic or inflammatory pathway for neuritis. An animal study showed that inflammation plays a key role in the pathogenesis of LNB [32]. Also, a recent study of 372 patients with acute peripheral facial palsy reported no difference in outcome of palsy due to LNB compared to subjects without LNB [33].

In our cohort of LNB patients, facial nerve enhancement has a strong correlation with clinical palsy. There are conflicting reports on the value of MRI as a prognostic marker in idiopathic facial neuritis—one study reported no association between intensity of enhancement and degree of palsy or outcome, while others found correlation between enhancement in IAC and presence of palsy or poor outcome [29, 34, 35]. We have not found previous studies of facial palsy that included the parotid segment in analyses. Our study of LNB patients shows that enhancement of the parotid segment is more specific, but less sensitive, than enhancement of distal IAC segment in predicting palsy at baseline and after 6 months. We found that 6 of 21 nerves with parotid segment enhancement had corresponding persistent palsy after 6 months compared to 6 of 48 with distal IAC segment enhancement. An important potential implication that is unstudied in idiopathic facial palsy is that parotid segment enhancement seems to increase the risk of poor outcome.

Still, a mild or moderate degree of enhancement in the distal IAC segment was not accompanied by palsy in 19 out of 37 nerves with this enhancement rate—more than can be attributed to possible spontaneous or physiological enhancement [11, 12]. This implies that subclinical facial neuritis in LNB is not uncommon, and is comparable to the finding in a study of idiopathic palsy where 30.8% had enhancement of the non-paralyzed nerve [36].

### Results for other cranial nerves

Oculomotor and abducens nerve enhancement mostly occurs with no evident eye movement palsy (16/17). A study from 2017 reported 7% with eye movement palsy in LNB [7] compared to 1.4% in our sample. The study with higher prevalence only included hospitalized patients, and therefore, probably a selected cohort of more severely affected patients who are not representative for all LNB patients. We found a low rate of enhancement of cranial nerves V (7/138, 5%), and VIII–XII (4/138, 3%), and little or no association with clinical manifestations.

### Study strengths and limitations

This is the first prospective study of a large cohort of LNB patients using reliable imaging ratings. We consider our study sample to be highly representative for LNB patients due to a prospective study design with a high rate of participation among patients treated in- and outside of hospital, and the proportion of subjects with a definite versus a possible LNB diagnosis is in line with other studies [5, 37]. Interobserver agreement of enhancement ratings for the important nerve locations is good, as in several other studies using a similar qualitative visual assessment for nerve enhancement [11, 35, 38]. Generalization of the findings concerning outcome may be limited by the subjects undergoing slightly different treatments, but all subjects received adequate treatment and a previous randomized clinical trial found oral doxycycline to be as efficient as intravenous ceftriaxone [23]. Subjects were scanned on two different MRI scanners, but we regard this to be a minor limitation as the scan protocol was set up as similar as possible, and two study centers gave a higher inclusion rate. This was a sub study, and the MRI protocol design was tailored to other objectives in addition to the cranial nerves. A dedicated study may benefit from smaller field of view and a matched healthy control group to provide information on physiological enhancement specific to the imaging protocol and type of contrast agent.

### Clinical implications

Knowledge that cranial nerve enhancement is common and often subclinical in LNB patients is valuable in the clinical setting. It can enable an effective and precise clinical work-up with cerebrospinal fluid analysis that includes looking for pleocytosis and *Bb* specific antibodies. Dismission of the enhancement as physiological or spontaneous in absence of palsy, or extensive search for neoplastic or inflammatory cause, may delay diagnosis and treatment. The finding of cranial nerve enhancement without corresponding clinical symptoms is not specific for LNB, but has also been described in idiopathic facial palsy [36] and multiple sclerosis [39]. For patients with facial palsy, extending evaluation of facial nerve enhancement to the extracranial parotid segment can add prognostic value: No parotid enhancement strengthens the expectation of normalized function within 6 months.

## Conclusion

In patients with pathological cranial nerve enhancement, Lyme neuroborreliosis should be considered a differential diagnosis regardless of whether clinical manifestations of neuropathy are present or not. For the facial nerve, emphasis should be on evaluation of the distal IAC and parotid nerve segments due to correlation with grade of palsy. Parotid segment enhancement seems to increase the risk of persistent palsy after 6 months.

## Supplementary Information

Below is the link to the electronic supplementary material.

Supplementary file1 (PDF 46.3 KB)

## Data Availability

The data that support the findings of this study are available on request from the corresponding author. The data are not publicly available due to privacy or ethical restrictions.

## Declarations

All procedures involving human participants in the study were in accordance with the ethical standards of the regional research committee and with the 1964 Helsinki Declaration and its later amendments or comparable ethical standards. The Regional ethics committee (South-Eastern Norway Health Authority) and Institutional data protection services approved of the study. Participants gave written informed consent.
