# Supplementary material for: Enhancement of cranial nerves in Lyme neuroborreliosis: incidence and correlation with clinical symptoms and prognosis
Source: Neuroradiology. 2022 May 24;64(12):2323–33. doi: 10.1007/s00234-022-02957-2 (PMC9643208; doi:10.1007/s00234-022-02957-2)
Supplement: Supplementary file 1 — Supplementary file1 (PDF 46.3 KB) [file 234_2022_2957_MOESM1_ESM.pdf]

Supplemental tables for article in Neuroradiology:

Enhancement of cranial nerves in Lyme neuroborreliosis: Incidence and correlation with clinical symptoms and prognosis

Authors:

Elisabeth S. Lindland MD[1,2,3], Anne Marit Solheim MD[4,5], Muhammad Nazeer Dareez MD[1],

Randi Eikeland MD, PhD[6,7], Unn Ljøstad MD, PhD[4,5], Åse Mygland MD, PhD[4,5,8], Harald Reiso MD, PhD[6], Åslaug R. Lorentzen MD, PhD[4,6], Hanne F. Harbo MD, PhD[3,9], Mona K. Beyer MD, PhD[2,3]

Affiliations:

[1]Department of Radiology, Sorlandet Hospital, Sykehusveien 1, N-4809 Arendal, Norway, [2]Division of Radiology and Nuclear Medicine, Oslo University Hospital, Oslo, Norway [3]Institute of Clinical Medicine, University of Oslo, [4]Department of Neurology, Sorlandet Hospital, [5]Institute of Clinical Medicine, University of Bergen, [6]The Norwegian National Advisory Unit on Tick-borne Diseases, Sorlandet Hospital, [7]Faculty of Health and Sport Sciences, University of Agder, [8]Department of Habilitation, Sorlandet Hospital, [9]Department of Neurology, Oslo University Hospital.

Corresponding author: e.m.s.lindland@studmed.uio.no

Supplemental table 1. Inter-rater statistics. The kappa statistic is paradoxical for several of the nerve locations due to a very low prevalence of some enhancement categories

Cranial nerve (segment)	Cohen's weighted kappa (95 % CI)	Percentage of agreement
**III (cisternal)**	0.80 (0.71-0.89)	85.5
**IV (cisternal)**	N/A[b]	97.1
**V (cisternal)**	0.71 (0.49-0.93)	93.5
**VI (cisternal)**	0.65 (0.48-0.82)	87.7
**VII (intraaxial)**	-0.11 (-0.23-0.002)	95.7
**VII (cisternal)**	N/A[b]	98.6
**VII (proximal IAC[a])**	N/A[b]	98.6
**VII (distal IAC[a])**	0.81 (0.75-0.87)	77.5
**VII (labyrinthine)**	0.64 (0.55-0.72)	63
**VII (geniculate ganglion)**	0.60 (0.47-0.74)	83.3
**VII (tympanic)**	0.23 (0.08-0.39)	76.1
**VII (mastoid)**	0.21 (0.09-0.68)	95.7
**VII (parotid)**	0.82 (0.73-0.91)	89.1
**VIII (cisternal)**	0.39 (0.09-0.68)	95.7
**IX-XI (cisternal)**	N/A[b]	96.4
**XII (cisternal)**	0.80 (0.48-1.11)	98.6

[a]IAC – internal auditory canal

[b]not executed because all ratings the same for at least one rater

Supplemental table 2. Correlation (Goodman and Kruskal's gamma) between facial palsy grade at baseline and at 6 months, and the acute phase enhancement rate for the facial nerve segments

Facial nerve segment	G (baseline)	p-value	G (6 months)	p-value
Intraaxial	-1.0	0.317	-1.0	0.348
Cisternal	-0.15	0.733	-1.0	0.107
Proximal IAC[a]	-1.0	0.156	-1.0	0.209
Distal IAC[a]	0.947	<0.001	1.000	0.008
Labyrinthine	0.962	<0.001	0.891	0.010
Geniculate ganglion	0.905	<0.001	0.917	0.025
Tympanic	0.947	0.009	0.937	0.148
Mastoid	0.711	0.010	0.971	0.101
Parotid	0.928	<0.001	0.982	0.008

[a]IAC – internal auditory canal
